# Clinical effects of cocktail injection on the thoracolumbar fascia injury during percutaneous vertebroplasty for osteoporotic vertebral compression fractures: a single-center, retrospective case-control study

**DOI:** 10.1186/s12891-023-07130-1

**Published:** 2024-01-02

**Authors:** Xiaolei Liu, Qinqin Zhou, Zhongyi Sun, Jiwei Tian, Haibin Wang

**Affiliations:** 1 Department of Orthopedics, the Fourth Affiliated Hospital of Nanjing Medical University, Nanpu road 298#, Jiangbei new District, Nanjing, 210000 China; 2 Department of Anesthesiology, the BenQ Hospital affiliated to Nanjing Medical University, Nanjing, 210000 China; 3 Department of Orthopedics, the BenQ Hospital affiliated to Nanjing Medical University, Nanjing, 210000 China

**Keywords:** Percutaneous vertebroplasty, Cocktail injection, Osteoporotic vertebral compression fractures, Thoracolumbar fascia injury, Residual pain

## Abstract

**Background:**

Nowadays, there is a lack of effective intraoperative treatment for thoracolumbar fascia injury (TFI) of osteoporotic vertebral compression fractures (OVCFs), which may lead to postoperative residual pain. We aimed to evaluate the clinical effects of cocktail injection on the TFI during percutaneous vertebroplasty (PVP) for OVCFs.

**Methods:**

A retrospective study of OVCFs with TFI underwent PVP with cocktail injection (Cocktail group, 58 cases) or PVP (Routine group, 64 cases) was conducted. The surgical outcomes, visual analog scale (VAS) score, oswestry disability index (ODI), incidence of residual pain at 1 day and 7 days postoperatively, the rate and duration of taking painkillers during 7 days postoperatively after PVP were compared between them.

**Results:**

No differences in baseline data, volume of bone cement injected and bone cement leakage were observed between the two groups, while the operation time of the routine group (44.3 ± 7.8 min) was less than that (47.5 ± 9.1 min) of the cocktail group (*P* < 0.05). However, the VAS scores (2.4 ± 0.8, 2.2 ± 0.7), ODI (25.2 ± 4.2, 22.3 ± 2.9), the incidence of residual pain (8.6%, 3.4%) at 1 and 7 days postoperatively, the rate (6.9%) and duration ( 2.5 ± 0.6 ) of taking painkillers during 7 days postoperatively in the cocktail group were better than those (3.4 ± 1.0, 2.9 ± 0.7, 34.1 ± 4.7, 28.6 ± 3.6, 23.4%, 15.6%, 28.1%, 4.2 ± 1.4) in the routine group (*P* < 0.05), respectively.

**Conclusion:**

PVP combined with cocktail injection increased the operation time in the treatment of OVCFs with TFI, but it can more effectively relieve pain, reduce the risk of residual pain at 1 day and 7 days postoperatively, and decrease the use and duration of taking painkillers.

## Introduction

The extension of human lifespan has led to a global trend of aging, which has resulted in an increased prevalence of osteoporosis [1, 2]. Osteoporosis is a chronic metabolic disease characterized by increased bone fragility and susceptibility to osteoporotic vertebral compression fractures (OVCFs) [3]. Given the higher mortality rate of individuals with OVCFs compared to the general population [4, 5], this condition has emerged as a significant global health concern for the elderly [6]. Percutaneous vertebroplasty (PVP) has emerged as a preferred treatment modality among orthopedic surgeons for alleviating pain and disability associated with OVCFs owing to its minimally invasive nature [7, 8].

However, a small subset of patients experienced residual pain after surgery due to inadequate symptom relief. Thoracolumbar fascia injury (TFI) has been identified as a significant risk factor for residual pain after PVP [9–14], which subsequently reduced surgical satisfaction. Anatomical and biomechanical studies have confirmed that the thoracolumbar fascia is an essential organizational structure of the spine and plays an indispensable role in spinal motion [15–18]. A recent study by Deng et al. [11] reported that TFI is regarded as the important concomitant damage in OVCFs patients and may lead to severe lower back pain. In cases where elderly patients experience residual pain due to TFI, conservative treatment methods such as oral analgesics and physical therapy are often employed post-surgery, which may prolong the treatment duration, impede postoperative rehabilitation exercises and increase the risk of gastrointestinal bleeding associated with painkillers [9, 11–13, 19].

Ultrasonic-guided erector spinae plane block is a commonly analgesic technique, which injects local anesthetics into the superficial thoracolumbar fascia and effectively reduces wound pain after lumbar surgery [20]. In addition, orthopaedic surgeons have innovatively proposed a cocktail injection to achieve the purpose of postoperative painless. Cocktail injection, based on local anesthetics and steroid hormones, has demonstrated favorable clinical outcomes in managing postoperative pain after total knee arthroplasty [21, 22]. However, there are no reports on the use of cocktail injection for thoracolumbar fascia to alleviate residual pain for TFI in OVCFs during PVP. Therefore, this study aimed to explore the clinical efficacy of combining PVP with cocktail injection for treating OVCFs with TFI.

## Methods

### Patient characteristics

The study was performed according to the Helsinki Declaration and was approved by the ethics committee of the fourth affiliated hospital of Nanjing Medical University (Ethics approval number: 20,230,109-k082), and informed consent was waived by the the ethics committee of the fourth affiliated hospital of Nanjing Medical University due to the retrospective nature of this study. A retrospective study of OVCFs with TFI from February 2019 to June 2021 was conducted. According to whether the cocktail injection for TFI was performed during PVP, the patients were divided into 64 cases of routine group (only PVP) and 58 cases of cocktail injection group (PVP combined with cocktail injection).

**Fig. 1:**
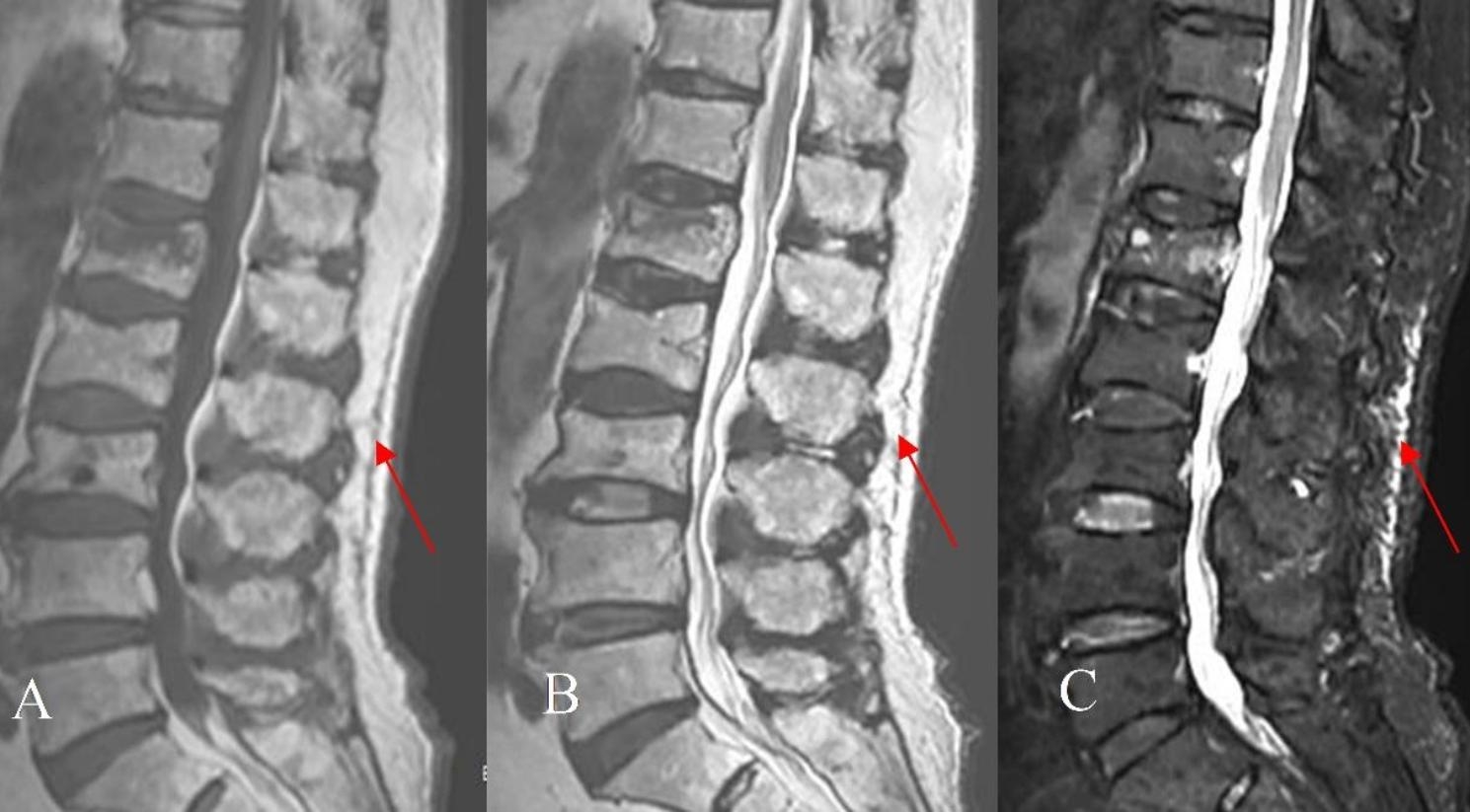
OVCF in the L1 vertebrae with TFI (Red arrows). MRI: the T1- weighted image **(A)** shows low signal intensity, and the T2- weighted image **(B)** and T2-weighted fat suppression image **(C)** show high signal intensity

The inclusion criteria were as follows: (1) Single-segment fresh OVCFs of T11-L4; (2) Combined with TFI diagnosised by Magnetic resonance imaging (MRI) of the T1-weighted images with low signal intensity, T2-weighted images with iso or high signal intensity and T2-weighted fat suppression images with high signal intensity (Fig. 1); (3) The range of TFI on sagittal MRI is approximately 1 to 4 vertebral heights; (4) No spinal cord injury; (5) The osteoporotic fracture (OF) classification of German Society for Orthopaedics and Trauma was OF1-OF2, and the score was ≥ 6 points [23, 24]; (6) T-value of bone mineral density was less than − 2.5; (7) Preoperative vertebral height loss was less than 1/3; (8) Good reduction of the vertebral body after operation; (9) Intolerable residual pain within 7 days after surgery are given only oral analgesics without other methods for pain controlled. The exclusion criteria were as follows: (1) Pathological fractures caused by tuberculosis, tumor and infection; (2) With intravertebral vacuum cleft, such as kummell’s disease; (3) With severe degenerative diseases of the spine; (4) Insufficient perfusion of bone cement injected; (5) Inability to operate due to serious diseases; (6) Poor distribution of intraoperative bone cement; (7) Patients with diabetes mellitus; (8) Patients with depression; (9) Allergic to ropivacaine and compound betamethasone; (10) Incomplete follow-up data.

The baseline data of the two groups was shown in Table 1. There were no significant differences in age, gender, bone mineral density, injured vertebral segment, fracture time, range of TFI, OF classification and score between the two groups (*P >* 0.05).

**Table 1:** Comparison of baseline data between the two groups

Variable	Routine group	Cocktail group	*P* value
Age (years)	69.5 ± 5.4	70.7 ± 5.7	0.238
Gender (female / male, n)	21 / 43	16 / 42	0.531
Bone mineral density (T score)	3.7 ± 0.5	3.8 ± 0.5	0.459
Injured vertebral segment (T11-L4, case)	9 / 15 / 19 / 14 / 4 / 3	7 / 18 / 20 / 8 /3 / 2	0.820
Fracture time (d)	5.3 ± 2.3	5.1 ± 2.6	0.693
OF classification (I / II, case)	11 / 53	8 / 50	0.606
OF score (point)	7.9 ± 1.2	8.0 ± 1.1	0.538
Range of TFI (mm)	51.6 ± 12.9	53.2 ± 14.4	0.504

### Surgical procedures

Routine group: bilateral transpedicle approach was performed. The patients were in the prone hyperextension position. The projection points of the pedicle on both sides of the injured vertebra were located and marked by C-arm X-ray machine. After disinfection and local anesthesia, incisions about 5 mm in length were taken at each projection point. Then, two puncture needles were inserted into the bilateral pedicles and reached the vertebral body guided by C-arm X-ray machine. Finally, the bone cement was slowly injected into the vertebral body under the guidance of C-arm X-ray machine. Attention should be paid to bone cement leakage during bone cement perfusion. When the bone cement had solidified, the incision was covered with gauze.

**Fig. 2:**
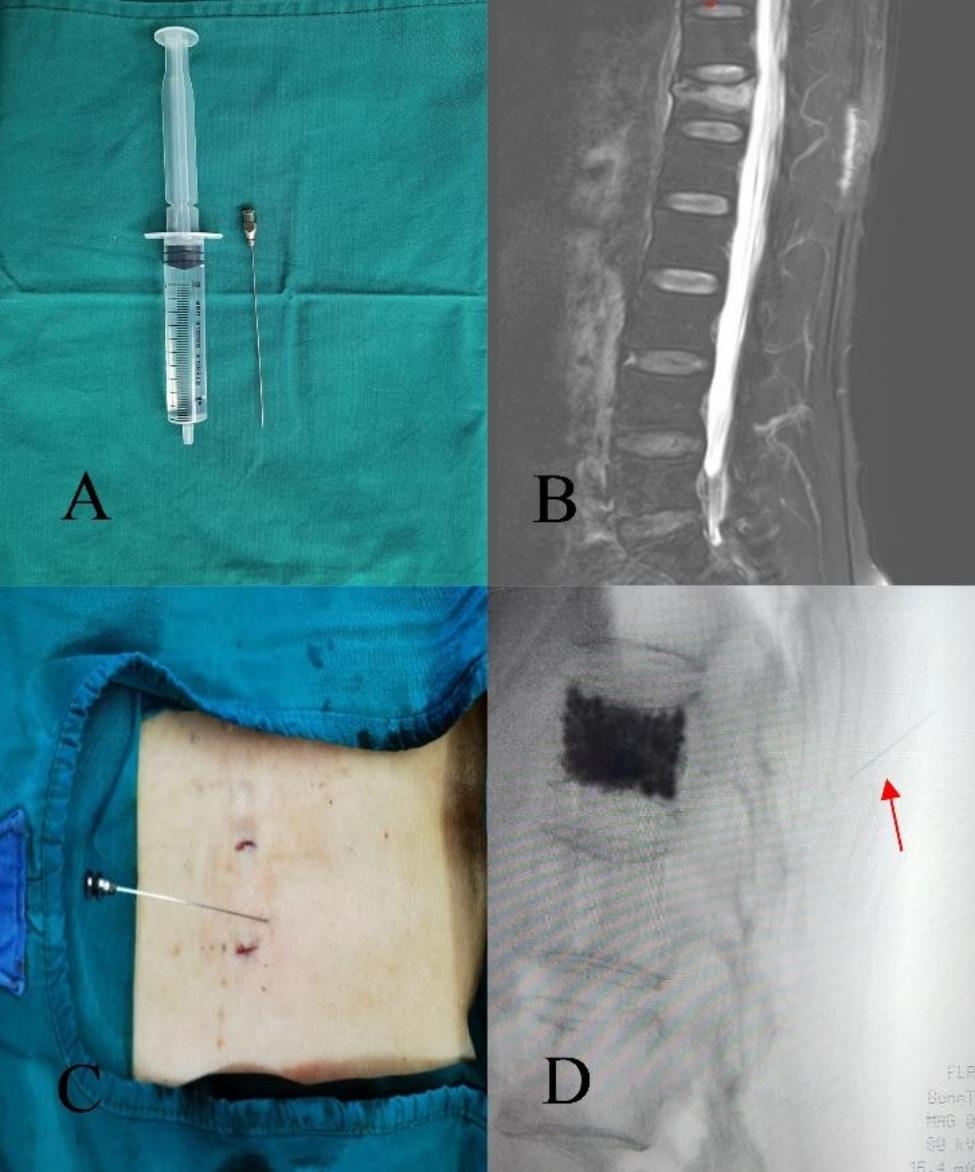
A 22-gauge needle and a 10 ml syringe **(A)** were applied to inject the cocktail; MRI of T2-weighted fat suppression image **(B)** showed T12 vertebral compression fracture with TFI; The needle guided by the C-arm X-ray machine **(C, D)** was used to locate and inject the cocktail in the area of TFI after PVP (The red arrow represented the needle)

Cocktail injection group: PVP was the same as routine group. The drug formula of cocktail was ropivacaine and compound betamethasone. Based on the length of the TFI on the sagittal MRI, the range of TFI equivalent to one vertebral body height is injected with a certain proportion of the cocktail, which includes 10 ml 0.25% ropivacaine and 0.25 ml compound betamethasone. The cocktail was extracted with a 10 ml syringe and injected with a 22-gauge needle (Fig. 2A). According to the location of TFI indicated by MRI (Fig. 2B), a 22-gauge needle was applied for locate the position under the guidance of C-arm X-ray machine (Fig. 2C, D) and a 10 ml syringe was uesed to inject the cocktail. During injection, the syringe should be drawn back to avoid entering the blood vessel.

### Postoperative management

All patients received standard anti-osteoporosis treatment. On the one hand, calcitriol capsules and calcium carbonate D3 were immediately used as the basal treatment after admission. On the other hand, zoledronic acid was used as the anti-osteoporosis drug after surgery. The patients got out of bed with waist brace for rehabilitation at 1 days postoperatively.

### Clinical assessment

Clinical indicators included the VAS score, ODI, incidence of postoperative residual pain, the rate and duration of painkillers taken during 7 days postoperatively. VAS score and ODI were used to evaluate the degree of pain and impact of life, respectively. The VAS score ≥ 4 was defined as residual pain [12]. When residual pain is intolerable during 7 days postoperatively, oral painkillers may be administered and duration of painkillers was recorded. 

### Statistical analysis

The SPSS26.0 statistical software was applied for data analysis. The quantitative data were expressed as mean ± standard deviation. When the data were normally distributed, the independent sample T test was used for inter-group comparison. The count data were expressed as frequency, and the Chi-square test or Fisher exact test was used for comparison between two groups. *P* value < 0.05 was considered statistically significant.

## Results

### Surgical outcomes

The surgical outcomes were summarized in Table 2. The operation was successful in both groups with no complications of spinal cord injury and pulmonary embolism related to bone cement leakage. In the cocktail group, there were no local anesthetic toxicity and complications such as hematoma and infection at the injection area. The average operation time of routine group was (44.3 ± 7.8) min, which was less than that (47.5 ± 9.1 min) of the cocktail group (*P* < 0.05). The average volume of bone cement injected was (5.6 ± 0.6) ml and 13 cases of bone cement leakage occurred in the routine group. While in the cocktail group, the average volume of bone cement injected was (5.7 ± 0.5) ml and bone cement leakage occurred in 10 cases. There were no statistical differences in the volume of bone cement injected and incidence of bone cement leakage between the two groups (*P* > 0.05).

**Table 2:** Comparison of surgical outcomes between the two groups

Variable	Routine group	Cocktail group	*P* value
Operation time (minute)	44.3 ± 7.8	47.5 ± 9.1	0.040
Volume of bone cement injected (ml)	5.6 ± 0.6	5.7 ± 0.5	0.352
Bone cement leakage (case, %)	13 (20.3%)	10 (17.2%)	0.665

### Clinical indicators

The comparison of clinical indicators between the two groups were presented on Table 3. The pain of both groups was significantly improved at 1 day postoperatively. The VAS score and ODI in the routine group and cocktail group decreased from (7.7 ± 0.7, 78.3 ± 5.1, 7.8 ± 0.8, 77.5 ± 4.6) at preoperation to (3.4 ± 1.0, 34.1 ± 4.7, 2.4 ± 0.8, 25.2 ± 4.2) at 1 day postoperatively and (2.9 ± 0.7, 28.6 ± 3.6, 2.2 ± 0.7, 22.3 ± 2.9) at 7 days postoperatively, respectively. The comparsions of preoperative VAS score and ODI between the two groups had no differences (*P* > 0.05). However, the VAS scores and ODI of cocktail group were better than those of routine group at 1 and 7 day postoperatively, respectively (*P* < 0.001). At 1 and 7 days postoperatively, there were 15 and 10 cases of residual pain in the routine group, while only 5 and 2 cases occurred in the cocktail group, and the comparison of them had statistical differences (23.4% vs. 8.6%, 15.6% vs. 3.4%, *P* < 0.05). During 7 days postoperatively, the rate and duration of taking painkillers due to residual pain in the routine group were higher than those in the cocktail group (28.1% vs. 6.9%, 4.2 ± 1.4 vs. 2.5 ± 0.6, *P* < 0.05).

**Table 3:** Comparison of clinical indicators between the two groups

Variable	Routine group	Cocktail group	*P* value
Preoperation			
VAS (score)	7.7 ± 0.7	7.8 ± 0.8	0.663
ODI (%)	78.3 ± 5.1	77.5 ± 4.6	0.351
1 day postoperatively			
VAS (score)	3.4 ± 1.0	2.4 ± 0.8	< 0.001
ODI (%)	34.1 ± 4.7	25.2 ± 4.2	< 0.001
Residual pain (case, %)	15 (23.4%)	5 (8.6%)	0.027
7 days postoperatively			
VAS (score)	2.9 ± 0.7	2.2 ± 0.7	< 0.001
ODI (%)	28.6 ± 3.6	22.3 ± 2.9	< 0.001
Residual pain (case, %)	10 (15.6%)	2 (3.4%)	0.024
Painkillers taken (case, %)	18 (28.1%)	4 (6.9%)	0.002
Duration of painkillers taken (day)	4.2 ± 1.4	2.5 ± 0.6	0.030

## Discussion

Anatomically, the thoracolumbar fascia can be divided into three layers, and common TFI refers to the superficial fascia, which is defined as injury based on MRI findings of fascia edema [9, 11–13]. Superficial thoracolumbar fascia is a complex arrangement of layers of fascia covering the surface of the erector spinal muscle of the trunk, which maintains the biomechanical stability of the thoracolumbar and participates in the movement of the thoracolumbar [15–18]. The paravertebral muscle is innervated by the dorsal branch of the spinal nerve. Therefore, the thoracolumbar fascia contains a rich distribution of sensory nerve endings [12, 16]. When the fascia is injured, it is easy to cause low back pain, which is also the anatomical basis of pain caused by TFI [25, 26].

It has been concluded that OVCFs with TFI are relatively common, particularly in elderly patients [11, 12], which is reported that the incidence of it was about 27.8% [11]. However, some orthopedic surgeons solely concentrate on OVCFs treatment and neglect the appropriate management of TFI during PVP [11]. Based on this phenomenon, these patients often experience residual pain due to inadequate postoperative pain relief, which not only prolongs bed rest and necessitates analgesic use but also impedes rehabilitation and reduces the postoperative satisfaction [9, 11–13]. Hence, it holds immense significance to investigate the efficacious management of OVCFs with TFI.

Residual pain is one of the common complications after PVP, which has been reported not rare [9, 12, 13]. Many studies have demonstrated that the occurrence of postoperative residual pain is associated with intravertebral vacuum cleft, TFI, poor distribution of bone cement, insufficient perfusion of bone cement, multiple segment PVP and depression [9–14]. Additionally, bone cement leakage and bone mineral density have also been considered as the risk factors of residual pain [14]. In order to minimize potential confounding factors, cases with intravertebral vacuum cleft, depression, bone cement underperfusion and poor bone cement distribution were excluded, and cases with single-segment fresh OVCF and good reduction of the vertebral body after operation were included in this study. Besides, there were no differences in range of TFI, bone cement leakage and bone mineral density between the two groups. At the same time, Patients with residual pain within 7 days after surgery were only treated with oral analgesics and had no other treatment options such as physical therapy in order to reflect the real VAS score, the rate and duration of taking painkillers. Therefore, it was further confirmed that the different treatment of TFI of two groups resulted in varying clinical outcomes. The findings of this study demonstrated that patients in the routine group exhibited significantly worsen VAS scores, ODI, and higher residual pain incidence compared to those in the cocktail group at 1 day and 7 days postoperatively.

To date, ultrasound-guided erector spinae block is to inject local anesthetics into the superficial thoracolumbar fascia to effectively relieve postoperative pain [20]. Based on this analgesic technique, orthopedic surgeons have innovatively proposed cocktail injection, including local anesthetics and steroid hormones, which is mainly applied in enhancing analgesia effects on wound pain after total knee arthroplasty, and has improved postoperative satisfaction [21, 22]. However, it is rarely used in PVP for OVCFs with TFI. In this study, the drug formula of cocktail was ropivacaine and compound betamethasone, which was precisely injected at the site of the TFI under the guidance of C-arm X-ray machine, and had obtained good clinical outcomes. Ropivacaine, as a long-acting local anesthetic [27], is characterized by separation of sensation and movement and can effectively block local peripheral nerves [28]. Besides, it is less toxic to the heart and central nervous system [29], and the concentration of it in this study is only 0.25%, which has greater safety [27, 29]. Compound betamethasone, as a long-acting steroid hormone, not only reduces the swelling of soft tissues, but also plays a huge anti-inflammatory effect with promoting the absorption of inflammatory cytokines. At the same time, it can delay the absorption rate of local anesthetics and prolong the analgesic time [30]. Therefore, the combination of them can more effectively relieve pain and decrease the use of painkillers, which this study has demonstrated.

PVP is a successful surgical procedure to treat OVCFs, which is usually performed under local anesthesia. The operation time of PVP is relatively short. Therefore, it has little influence on the functions of the heart, lung, brain and other important organs in elderly patients. It has shown that PVP is safe in the treatment of multi-segment OVCFs [31], even though the operation time of it is significantly longer than that of single-segment OVCFs. In this study, the operation time of the cocktail group was more than that of the routine group, but significantly less than that of the multi-segment OVCFs. Although the operation time of the cocktail group was prolonged, no serious complications occurred during the perioperative period.

Nonetheless, the study had several limitations. First, it was a single-center, retrospective and controlled study, which needed to be further validated by high-quality, multi-center, prospective and randomized studies. Second, the sample size in this study was small and a larger-sample is needed to reduce bias. Third, although common risk factors of residual pain were excluded in this study, there were still other interference factors such as the effects of degeneration of low back muscles on residual pain. Finally, this study did not make the comparison of the long-term effects. Therefore, we will remove the above deficiencies to improve this study in the future.

## Conclusion

In conclusion, PVP combined with cocktail injection in the treatment of OVCFs with TFI may increase the operation time, but it can more effectively relieve pain, reduce the risk of residual pain at 1 day and 7 days postoperatively, and decrease the use and duration of painkillers taken.

## Data Availability

Data and materials included in the study are available from the corresponding author on request.
